# Causes and characteristics of medical student referrals to a professional behaviour board

**DOI:** 10.5116/ijme.584b.d591

**Published:** 2017-01-15

**Authors:** Pieter C. Barnhoorn, Jan H. Bolk, Marleen W. Ottenhoff- de Jonge, Walther N.K.A. van Mook, Arnout Jan de Beaufort

**Affiliations:** 1 Department of Public Health and Primary Care, Leiden University Medical Centre, The Netherlands; 2 Department of Intensive Care Medicine, Maastricht University Medical Centre, The Netherlands

**Keywords:** Professional behaviour, professionalism, professional identity formation, medical students, professional behaviour board

## Abstract

**Objectives:**

To describe the nature of unprofessional behaviour displayed by medical students, as well as the characteristics of students referred to the professional behaviour board.

**Methods:**

A descriptive mixed methods approach was taken, in which qualitative data on unprofessional behaviour, as well as quantitative data on the demographics of referred students were collected during the study period between June 1, 2009 and January 1, 2014. In order to compare the referred students with the total student population, data on gender, nationality and phase in the curriculum of the total student population, collected from the student administration desk, were also used.

**Results:**

In the study period, a total of 107 referrals were reported, concerning 93 different students (3% of the total student population). Sixty-five of the 107 referrals (61%) concerned male students. Thirty referrals (28%) concerned non-Dutch students. Most referrals (71%) occurred during clinical rotations. The referrals were equally distributed over three professional behaviour domains: dealing with oneself, dealing with others, and dealing with tasks/work. ‘Withdrawn behaviour’ was reported 17 times, ‘insufficient Dutch language proficiency’ 14 times, ‘impertinent emails’ 9 times and ‘placing privacy-sensitive photos on the internet’ 3 times.

**Conclusions:**

Although only a minority of students are referred to a professional behaviour board, this study shows that student characteristics such as gender and nationality may correlate to a higher incidence of unprofessional behaviour. Further explanatory and exploratory research is needed to unravel this relationship, and to study the influence of curriculum reforms on these relationships, respectively.

## Introduction

Over the past three decades, professionalism has been incorporated as a major theme in most medical curricula.^1^^-^^3^ Professionalism is a key competence in the CanMEDS 2015 framework used in both undergraduate and postgraduate medical education programmes.^4^ In addition, several studies report that students’ unprofessional behaviour at medical schools often precedes disciplinary action by medical boards as physicians.^5^^,^^6^ Parallels have also been found between the type of problematic behaviour in students and practising physicians.^7^

Until recently, two lines of thought on professionalism could be distinguished: professionalism as a trait, a personal characteristic or a feature of a student that is susceptible to development but hard to assess, and professional behaviour as the actual observable behaviour that can be assessed.^8^ Recently, the concept of ‘Professional Identity Formation’ (PIF) has been added to the discourse.^9^^-^^15^ PIF describes the process by which students are transformed from members of the lay public into skilled professionals.^12^^,^^13^ In this process, the student’s underlying attitudes and virtues become increasingly visible in the student’s actual (professional) behaviour. However, few tools are currently available to assess a student’s progress towards the formation of a professional identity.^14^^,^^15^ Even when acknowledging that professionalism encompasses more than just behaviour,^16^ as the literature on PIF shows, a focus on unprofessional behaviour is still indispensable.^17^ Knowledge of  the exact nature of unprofessional behaviour and the characteristics of students who behave unprofessionally can help to guide curriculum development, faculty development, the development of remediation strategies and future research.^18^ Thus far, many questions regarding the extent of occurrence, the exact nature and the risk factors of unprofessional behaviour among future doctors remain.^18^ Similar observations led Papadakis and colleagues to describe their research agenda for medical professionalism: they expressed the need to gather new evidence regarding demographics, ethnographics, and the epidemiology of lapses in professional behaviour.^19^ Prior to initiating tailor-made remediation practices for unprofessional behaviours, the exact nature of the unprofessional behaviour and the possible underlying causes should be studied. However, in many articles published on professionalism, the exact nature of unprofessional behaviour and student characteristics are rarely explicated.^18^^,^^20^^,^^21^ This study aims to describe the nature of the unprofessional behaviour displayed, as well as the characteristics of students referred to the professional behaviour board.

### Context of the study

The current definition of professional behaviour used in all Dutch medical schools is ‘the observable behaviour from which the norms and values of the medical professional can be visualised’. Professional and unprofessional behaviour is divided into three domains: dealing with oneself, dealing with others, and dealing with tasks/work (Table 1).^22^ At Leiden University Medical Center (LUMC), students who are judged to have behaved unprofessionally are referred to the professional behaviour board. The professional behaviour board, established in 2009, consists of 6 physicians, 1 midwife and 1 psychologist, all of whom are closely involved in the education of medical students. Lecturers, medical and paramedical personnel, as well as students’ peers can refer a student to the professional behaviour board during his/her bachelor’s and master’s programme. The board discusses the referrals every fortnight and invites referred students to engage in debate with two members of the board. Subsequently, the board provides ‘tailor-made’ advice: a student may be advised, for example, to seek psychological support or to suspend his/her studies.

## Methods

### Study design

To gain more insight into the exact nature of the unprofessional behaviour displayed, as well as into the characteristics of referred students, a descriptive mixed methods approach was chosen. Qualitative data on the displayed unprofessional behaviour were collected from both the referral letters and the dossiers of the professional behaviour board. Quantitative data about the demographics of students were collected from the professional behaviour board dossiers and the student administration desk.

### Participants

The total group of medical students enrolled in the bachelor’s or master’s programme at Leiden University during the study period between June 1, 2009 and January 1, 2014 consisted of 3410 students. In this study period, the board received 107 referrals concerning 93 students (3% of a total of 3410 students). Eight students were referred twice, and 2 students were referred four times.

Consultation of the Medical Ethics Review Committee of the Leiden University Medical Center revealed that ethical approval was not needed for this study given the nature of the research, as all data were solely accessible by members of the professional behaviour board. The researchers who analysed the referral letters (PB and JB) are both members of the professional behaviour board.

**Table 1:** Domains of professional behaviour and examples

Domains of professional behaviour	Examples
Dealing with oneself	Self-reflection: the student is able to reflect on his/her self; is able to detect weak spots and is able to formulate learning objectives Feedback: the student is open-minded when faced with criticism and the right criticism does change his/her behaviour Appearance: the student looks appropriate with regard to clothing and hygiene during study meetings and when dealing with patients Self-management: the student knows his/her tasks and is able to set boundaries
Dealing with others	Verbal communication: the student communicates proficiently in Dutch language Nonverbal communication: the student uses adequate nonverbal language Cooperation: the student can act well in a group of staff, peers and patients Privacy: the student protects confidential information about patients and colleagues
Dealing with tasks/work	Responsibility: the student shows responsibility in studying and in dealing with patients Time-management: the student performs his/her tasks in an efficient way Independence: the student is able to make assignments or deal with patient encounters without too much support Performance: the student is conscientious and punctual

### Data collection

Data on gender, curricular phase when the referral was made, and nationality of the referred students were collected from the student administration desk. As only the country of birth of the students is registered, students were classified as non-Dutch if the official language of the country of birth on record was a language other than Dutch.

Data on the reasons for referral and the number of referrals per student were collected from referral letters and professional behaviour board dossiers.

In order to compare the referred students with the total student population, data on gender, nationality and phase in the curriculum of the total student population were also collected from the student administration desk.

### Data analysis

First, one of the researchers (PB) analysed all referral letters, the professional behaviour board dossiers, and the distinct codes attached to every referral during the fortnightly meetings of the board. This code consists of the shortest possible summary of the reason for referral, recorded in the minutes. Hereafter, he categorised the reasons for referral into one or more of the domains of professional behaviour, according to the classification used in The Netherlands (dealing with oneself, dealing with others, and dealing with tasks/work; see Table 1).^22^^-^^24^ As the number of codes grew, the on-going process of renaming and reorganising codes resulted in a grouping of codes by themes. To monitor for inter-coder agreement another member of the research team (JB) independently repeated the categorisation for the first 2 years. The inter-coder agreement was found to be very high. Data on gender, nationality and curricular phase of the referred students were compared with the total student population.

## Results

In the study period, the board received 107 referrals concerning 93 students (3% of a total of 3410 students). Eight students were referred twice, and 2 students were referred four times.

### Characteristics of referred students

Sixty-five of the 107 referrals (61%) concerned male students. The distribution of male versus female at our medical school was fairly constant during the study period (65% female). The odds of referral were 2.98 times higher for male students as compared to female students.

Thirty of the 107 referrals (28%) concerned non-Dutch students. The percentage of students at our medical school who were born outside the Netherlands was relatively constant over the last four years at between 12% and 13%. The odds of referral were 2.86 times higher for non-Dutch students compared to Dutch students.

Seventy-six referrals (71%) occurred during the clinical rotations (Table 2).

### Nature of the unprofessional behaviour

Sixty-five referrals concerned the domain ‘dealing with others’ (61%), followed by the domain ‘dealing with oneself’, which saw 53 referrals (50%). In 49 cases (46%) the domain ‘dealing with tasks’ was at issue. For 56 referrals (52%), the unprofessional behaviour consisted of a combination of two or three domains.

In the process of categorising the reasons for referral, four types of behaviour were recorded more than twice: ‘withdrawn behaviour’ (17 times), ‘insufficient Dutch language proficiency’ (14 times), ‘impertinent emails’ (9 times), and ‘placing (privacy-sensitive) photos of the dissection room on the internet’ (3 times) (see Table 2).

**Table 2:** Characteristics of the students referred to the professional behaviour board (N=107)

Demographics	Referrals N (%)
Female/Male	42/65 (39%/61%) Total student population 65%/35%
Non-Dutch/Dutch	30/77 (28%/72%) Total student population 12%/88%
Pre-clinical/Clinical	31/76 (29%/71%) Total student population 50%/50%
Domains	
dealing with others	65 (61%)
dealing with tasks	49 (46%)
dealing with self	53 (50%)
combinations of domains	56 (52%)

## Discussion

This study is the first to analyse medical student referrals to a professional behaviour board in detail. It is also one of the few studies to describe the exact nature of concrete, observed unprofessional behaviour.^18^ The findings that men, non-Dutch minorities, and referrals from the clinical workplace dominate are pivotal for further research. Predominant reasons for referral such as ‘withdrawn behaviour’, ‘insufficient Dutch language proficiency’, ‘impertinent emails’, and ‘placing privacy-sensitive photos on the internet’ have already resulted in an adjustment of our information policy, in addition to specific remediation. Special effort should be made to clarify what faculty expects from students and teachers.^25^ The observations in this study can potentially guide future research, curriculum and faculty development and the development of remediation strategies. This will be elaborated in the sections below and discussed in light of the available literature.

### Characteristics of referred students

#### Male Students

Male students’ higher odds of getting referred raise the question of whether male students do indeed behave unprofessionally more often than female students do, or if a gender-specific bias exists in the likelihood of referral. Recent studies show gender differences in motivation, learning variables and learning outcomes.^26^^,^^27^ Male students have a significantly higher so-called ‘controlled motivation’ compared to female students.^26^ Controlled motivation represents motivation which is very low on self-determination. The more self-determined a student is, the better the observed outcomes with regards to deep learning, academic performance, adjustment, and well-being.^26^ Combined with the overrepresentation of male students among referrals, these findings raise the question of whether male students require different types of mentoring than female students.^26^ To answer this question, further (longitudinal) studies are needed.

#### Non-Dutch Students

Another group of overrepresented students were non-Dutch students, a minority at the LUMC. There are a number of possible explanations for the underperformance of minorities.^28^^,^^29^ When considering unprofessional behaviour to be a form of underperformance, some of these explanations may also explain the overrepresentation of non-Dutch students in our study. First, a deficit in practical clinical knowledge may exist in students who belong to an ethnic minority.^28^ Second, (cultural) differences in communication styles may be a possible explanation for these students’ underperformance.^28^ A third possible explanation is the so-called ‘stereotype threat’, which means that underperformance in ethnic groups could be caused by increased anxiety which arises in the student in response to the prospect of being negatively stereotyped.^28^ Finally, more subjective grading in clinical training can lead to what is called ‘examiner bias’, which means that examiners have a more positive view on people who are similar to themselves and who are believed to be a part of their own group than on people who do not to fall into those categories.^28^ In summary, Dutch examiners may mark Dutch students more highly than non-Dutch students for a variety of reasons. Future studies should be designed to address these hypotheses in the context of professional behaviour and medical education.

#### Clinical Rotations

The majority of referrals occurred during clinical rotations. Clinical rotations facilitate the observation of students and the assessment of their behaviour. However, it would be preferable to detect unprofessional behaviour earlier in a student’s career, which would provide more opportunities for remediation. That is why longitudinal professionalism training was introduced as part of our curricular revision in 2012. This training consists of small working group sessions in which professionalism and communication training are discussed, as well as individual mentoring sessions in which academic progress and, if necessary, professional lapses are discussed. The first students enrolled in the new curriculum entered their master’s programmes in June 2015. We are currently exploring how the new bachelor’s programme has influenced the number and specifics of the referrals to the professional behaviour board.

### Nature of the unprofessional behaviour

The referrals analysed are evenly distributed over the three domains dealing with oneself, dealing with others, and dealing with tasks/work. This subdivision in domains is useful to give the student more insight into the nature of their unprofessional behaviour, and will therefore guide remediation.

The remediation strategies of the four types of behaviour that were recorded more than twice will be described briefly. These four types of behaviour were ‘withdrawn behaviour’, ‘insufficient Dutch language proficiency’, ‘sending impertinent emails’ and ‘placing privacy-sensitive photos on the internet’.

### Withdrawn behaviour

Most students referred due to ‘withdrawn behaviour’ seemed to suffer from psychological problems such as depression or social anxiety disorders. These students were referred to a psychologist.

### Insufficient Dutch language proficiency  

The category ‘insufficient Dutch language proficiency’ consisted solely of non-Dutch students who could insufficiently express themselves in Dutch. These students were all advised to practice their Dutch or to take an intensive Dutch language course. More stringent selection criteria for admission to our medical school regarding Dutch language proficiency led to a decline in referrals for this reason.

### Impertinent emails

A study on professional behaviour online was set up among students and teachers, as ‘sending impertinent emails’ is a frequent reason for referral, and because teachers and students seem to disagree on what is considered an ‘impertinent email’. Based on the results of this study, a code of conduct for students has been created. This code will be addressed explicitly in a first-year lecture on professional behaviour.

### Privacy-sensitive photos

Placing privacy-sensitive photos on the internet has ceased to be a reason for referral, as this too is now explicitly addressed at the beginning of the study program.

### Limitations and future directions

This study has a number of limitations. First, the data were collected from only one institution, spanning a period of just 4.5 years, with a limited sample size (107 referrals from 93 different students). A study has already been set up into the nature of unprofessional behaviour and the characteristics of students who behave unprofessionally, using data from all eight Dutch medical schools. Preliminary data presented at meetings of the ‘Netherlands Association for Medical Education Professionalism Think Tank’ seem to reveal nationally comparable trends in the demographics and the epidemiology of lapses in professional behaviour.^30^

Another limitation of the current study is that not all referrals were documented in detail during the first years of the professional behaviour board. As teachers have since been trained in referring, nearly all referrals records are complete as of now.

Furthermore, there may be an underreporting of cases. In our study, 3% of all medical students were referred to the professional behaviour board, a figure which is comparable to other medical schools.^31^ However, as almost all cases reported undisputed unprofessional behaviour, it is likely that the referred 3% is only the tip of the iceberg. Teachers are indeed known to experience barriers in referring students.^31^^-^^33^ Unprofessional behaviour might be underreported for reasons similar to those of clinical lecturers who do not fail students. Dudek et al. identified four major barriers to failing trainees: ‘(1) lack of documentation, (2) lack of knowledge of what to specifically document, (3) anticipating an appeal process, and (4) lack of remediation options’.^34^ These possible reasons for underreporting should be taken into account for faculty development. Recent research by Mak-van der Vossen et al. indicates that supporting teachers in assessing professional behaviour and involving them in the remediation of unprofessional behaviour helps to overcome these barriers.^31^

Finally, for privacy reasons only the country of birth of students was available. In legal terms, a person is considered “Dutch native” (autochthonous) when both parents are born in the Netherlands. Students were classified as non-Dutch if the official language of their country of birth was a language other than Dutch. However, country of birth is not a perfectly accurate way of identifying non-Dutch students, which is why this study likely underreports the number of non-Dutch students.

## Conclusions

Knowledge of the exact nature of displayed unprofessional behaviour and the characteristics of students referred to a professional behaviour board is needed to guide curriculum development, faculty development, the development of remediation strategies and future research. Although only a minority of students is referred to a professional behaviour board, our study shows that student characteristics such as gender and nationality may correlate to a higher incidence of unprofessional behaviour. Further explanatory and exploratory research is needed to unravel this relationship, and to study the influence of curriculum reforms on these relationships, respectively.

### Conflict of Interest

The authors declare that they have no conflict of interest.
